# Brain oxygenation monitoring during neonatal stabilization and resuscitation and its potential for improving preterm infant outcomes: a systematic review and meta-analysis with Bayesian analysis

**DOI:** 10.1007/s00431-025-06138-0

**Published:** 2025-04-21

**Authors:** Marlies Bruckner, Thomas Suppan, Ena Suppan, Bernhard Schwaberger, Berndt Urlesberger, Katharina Goeral, Marlene Hammerl, Tina Perme, Eugene M. Dempsey, Laila Springer, Gianluca Lista, Tomasz Szczapa, Hans Fuchs, Lukasz Karpinski, Jenny Bua, Brenda Law, Julia Buchmayer, Ursula Kiechl-Kohlendorfer, Lilijana Kornhauser Cerar, Christoph E. Schwarz, Kerstin Gründler, Ilaria Stucchi, Katrin Klebermass-Schrehof, Georg M. Schmölzer, Gerhard Pichler

**Affiliations:** 1 Division of Neonatology, Department of Pediatrics and Adolescent Medicine, Medical University of Graz, Auenbruggerplatz 34/2, 8036 Graz, Austria; 2 Research Unit for Neonatal Micro- and Macrocirculation, Medical University of Graz, Graz, Austria; 3 Institute of Electrical Measurement and Sensor Systems, Graz University of Technology, Graz, Austria; 4 Comprehensive Center for Pediatrics, Department of Pediatrics and Adolescent Medicine, Division of Neonatology, Intensive Care and Neuropediatrics, Medical University of Vienna, Vienna, Austria; 5 Department of Pediatrics II, Medical University of Innsbruck, NeonatologyInnsbruck, Austria; 6 NICU, Department for Perinatology, Division of Gynaecology and Obstetrics, University Medical Centre Ljubljana, Ljubljana, Slovenia + Medical Faculty, University of Ljubljana, Ljubljana, Slovenia; 7 INFANT Research Centre, University College Cork, Cork University Maternity Hospital, Cork, Ireland; 8 Department of Neonatology, University Children’s Hospital of Tübingen, Tübingen, Germany; 9 Neonatologia E Terapia Intensiva Neonatale (TIN) Ospedale Dei Bambini “V Buzzi,” Milan, Italy Milan,; 10 II Department of Neonatology, Neonatal Biophysical Monitoring and Cardiopulmonary Therapies Research Unit, Chair of Neonatology, Poznan University of Medical Sciences, Poznan, Poland; 11 Division of Neonatology and Pediatric Intensive Care Medicine, Center for Pediatrics and Adolescent Medicine, Medical Center, Faculty of Medicine, University of Freiburg, University of Freiburg, Freiburg, Germany; 12 Institute for Maternal and Child Health, Neonatal Intensive Care Unit, “IRCCS BurloGarofolo,”, Trieste, Italy; 13 Centre for the Studies of Asphyxia and Resuscitation, Neonatal Research Unit, Royal Alexandra Hospital, Edmonton, AB Canada; 14 Department of Pediatrics, University of Alberta, Edmonton, AB Canada

**Keywords:** Neonatal resuscitation, Neonatal stabilization, Delivery room, Brain oxygenation, Cerebral tissue oxygen saturation, Near-infrared spectroscopy, NIRS, Preterm infants, Neonates, Bayesian

## Abstract

Neonatal stabilization and resuscitation in preterm infants are critical interventions. Cerebral tissue oxygen saturation (CrSO2) measured with near-infrared spectroscopy monitoring offers potential benefits by providing real-time information on brain oxygenation. This systematic review aimed to determine if CrSO2-monitoring to guide neonatal resuscitation after birth can improve survival without cerebral injury. A systematic search of MEDLINE, Google Scholar, EMBASE, the Cumulative Index of Nursing and Allied Health Literature, Clinical Trials.gov, and the Cochrane Central Register of Controlled Trials was performed through December 2024. We included only human studies that investigated CrSO2-guided interventions during neonatal resuscitation after birth in preterm infants. A meta-analysis was performed using individual patient data and the Bayesian method. The main outcome assessed was survival without cerebral injury (Study registration:PROSPERO CRD42024512148). Two studies were identified, including a total of 667 preterm infants with less than 34 weeks of gestation, describing CrSO2-guided interventions during neonatal resuscitation. The meta-analysis revealed a high probability of treatment superiority for NIRS-guided interventions that demonstrated improved outcomes compared to standard care, with a 4.5% increase in the rate of survival without cerebral injury (93% probability) and 4.2% reduction of IVH of any grade (94% probability). The risk of bias can be described as low.

Conclusion:This meta-analysis suggests that CrSO2-guided interventions may offer a meaningful advantage in preterm infant resuscitation after birth, improving survival without brain injury. The analysis indicates a high probability of a clinically important benefit. This warrants consideration in clinical practice.

**Table:** 

**What is Known:** • *Studies have shown that near-infrared spectroscopy can monitor brain oxygenation in preterm infants immediately after birth.*	
**What is New:** • *This is the first meta-analysis to examine the impact of near-infrared spectroscopy based interventions on neonatal resuscitation outcomes.* • *Interventions based on monitoring preterm infants' cerebral oxygenation may improve their chances of surviving without severe brain injury, compared to standard care.*

**Supplementary Information:**

The online version contains supplementary material available at 10.1007/s00431-025-06138-0.

## Introduction

Extremely low gestational age preterm infants, born less than 29 weeks’ gestation, have a high risk of mortality and cerebral injury, including intraventricular hemorrhage (IVH) and periventricular leukomalacia (PVL) (1–3)). The first minutes after birth are crucial for the preterm infant’s outcome, in addition to the first hours following delivery (4, 5)). Current neonatal resuscitation guidelines recommend monitoring preterm infants’ heart rate and arterial oxygen saturation (SpO_2_) using pulse-oximetry and optionally electrocardiogram (ECG) (6, 7)).

However, these conventional monitoring options do not directly provide information about brain oxygenation during the critical neonatal stabilization period. Interestingly, multiple studies have demonstrated the feasibility of using near-infrared spectroscopy (NIRS) to monitor neonatal brain oxygenation, even in extremely preterm infants, within the first minutes after birth (8–10)). This non-invasive NIRS technology allows for the assessment of cerebral tissue oxygen saturation (CrSO2), which can help to identify preterm infants with impaired brain oxygenation. The availability of published normative values for CrSO2 in preterm infants (11–14) further enables clinicians to recognize when brain oxygenation is outside the expected range, potentially prompting targeted interventions to optimize brain perfusion and oxygen delivery during this critical period.

Therefore, utilizing information about brain oxygenation during resuscitation and performing interventions when it is out of range could be a valuable approach to improve preterm infants’ outcomes. While there are published randomized controlled trials (RCT) focusing on this question, so far, no systematic review has been published describing the outcomes of those studies.

Bayesian statistical approaches are garnering increased attention as they can assist clinicians in more effectively translating research findings into clinical practice. Unlike traditional frequentist methods, which simply determine the presence or absence of an effect, Bayesian analysis describes the probability of an intervention’s effects. This might allow clinicians to better comprehend the likelihood of treatment benefits and make more informed clinical decisions (15).

The aim of this systematic review and Bayesian-analysis of individual patients’ data was to determine whether survival without cerebral injury was more probable using CrSO2 to guide interventions during neonatal resuscitation when compared to the standard monitoring methods (15)(15–17)(18).

## Methods

This review was conducted with the standard methods of Cochrane Handbook for Systematic Reviews of Interventions Version 6.5 (19)). Reporting was in accordance with the Preferred Reporting Items for Systematic Reviews and Meta-Analyses (PRISMA) (20)). The review has been submitted to the International Prospective Register of Systematic Reviews (PROSPERO CRD42024512148).

We searched the following electronic databases: MEDLINE, Google Scholar, EMBASE, the Cumulative Index of Nursing and Allied Health Literature, Clinical Trials.gov, and the Cochrane Central Register of Controlled Trials. We used a predefined algorithm with the search terms neonate, newborn, cerebral oximetry, cerebral regional oxygenation, near-infrared spectroscopy, NIRS, delivery room, resuscitation, transition, after birth, intervention, care, and treatment. Additionally, we performed a manual search of references in articles identified by our search strategy. No language or publication period restrictions were applied, and the search was performed through December 2024. Studies conducted in older patients or in settings outside the delivery room were excluded.

### Study and data selection

Two review authors (MB and ES) independently screened titles and abstracts assessed full-text articles for eligibility and resolved any disagreements through discussion. Any discrepancies regarding inclusion were resolved through consensus. Then, the full-text articles were retrieved and included based on the eligibility criteria. This systematic review included only RCTs involving preterm infants with a gestational age less than 37 weeks, who underwent resuscitation in accordance with current neonatal resuscitation guidelines and additionally received interventions based on CrSO2 measurements. The primary outcome was survival without cerebral injury. Cerebral injury was defined as IVH any grade and/or cystic PVL diagnosed at any moment until discharge. Secondary outcome parameters were the incidence of necrotizing enterocolitis, retinopathy of prematurity, and bronchopulmonary dysplasia.

### Data extraction

Data extraction was performed using a standardized data collection form that included study design, methods, patient characteristics, interventions, and outcomes. We used Microsoft Excel (Version 16, Microsoft Corporation, Redmond, Washington, USA) to document the mode of randomization, allocation concealment, blinding, and adherence to the intention-to-treat principle. Two independent investigators (MB and ES) extracted the data and resolved any discrepancies through discussion and additional review of the case report forms.

### Assessment of methodological quality and data synthesis

The methodological quality of the included trials was assessed using the risk of bias in randomized trials (RoB2) of the Cochrane Collaboration tool, which evaluated the risk of bias (21). The domains assessed included randomization, allocation concealment, blinding, and adherence to the intention-to-treat principle.

We aimed to obtain individual patient data of included studies, which allowed us to perform Bayesian analysis for the primary outcome, e.g., survival without cerebral injury (IVH any grade and/or cystic PVL) as well as for the secondary outcome parameters including mortality, IVH, PVL, necrotizing enterocolitis, retinopathy of prematurity, and bronchopulmonary dysplasia. Individual patient data were recoded into common format and checked for completeness of records, values, and variables, and internal consistency (i.e., out-of-range values), external consistency with published reports, compliance, validity, plausibility, and duplicate entries.

### Statistical analysis

We performed a Bayesian meta-analysis to evaluate the efficacy of interventions based on NIRS monitoring for survival without cerebral injury across two RCTs. A Bayesian hierarchical model was used to describe the posterior distribution of the probability to observe the primary and secondary outcomes. Hereby, the effect sizes of the individual studies are modelled as realizations of an overarching normal distribution. The mean corresponds to the pooled effect size and the variance describes the between study heterogeneity. However, due to the small number of studies included in the meta-analysis and since both RCT are direct replications and stem from the same center, the between-study heterogeneity was not evaluated and set to zero. This approach corresponds to the fixed effect model for meta-analyses (22, 23). A binomial distribution was used as a likelihood function to model the binary data, i.e., primary outcome observed, or primary outcome not observed, accommodating the number of events within the NIRS and control groups.

For the pooled effect size, a weakly-informative uniform prior was used. The uniform prior distribution allows the data to drive the estimates by restricting the probability for the primary outcome to feasible values between zero and one, without favoring particular values within this range. Since a uniform prior was used, a sensitivity analysis was deemed unnecessary. The posterior distribution for the primary outcome effect size was derived by combining the likelihood function and the prior distribution using Bayes’theorem for both, the standard care group as well as the NIRS-guided group.

The computation of the posterior distributions as well as posterior inference was carried out using numerical integration, implemented in Python. Posterior estimates were summarized by the posterior mean, 95% credible interval, and probability of effect in the direction of interest.

Posterior predictive checks were conducted to evaluate model fit, confirming that the observed data were well-represented by the posterior model. Detailed statistical report is available in supplementary material.

## RESULTS

**Fig. 1:**
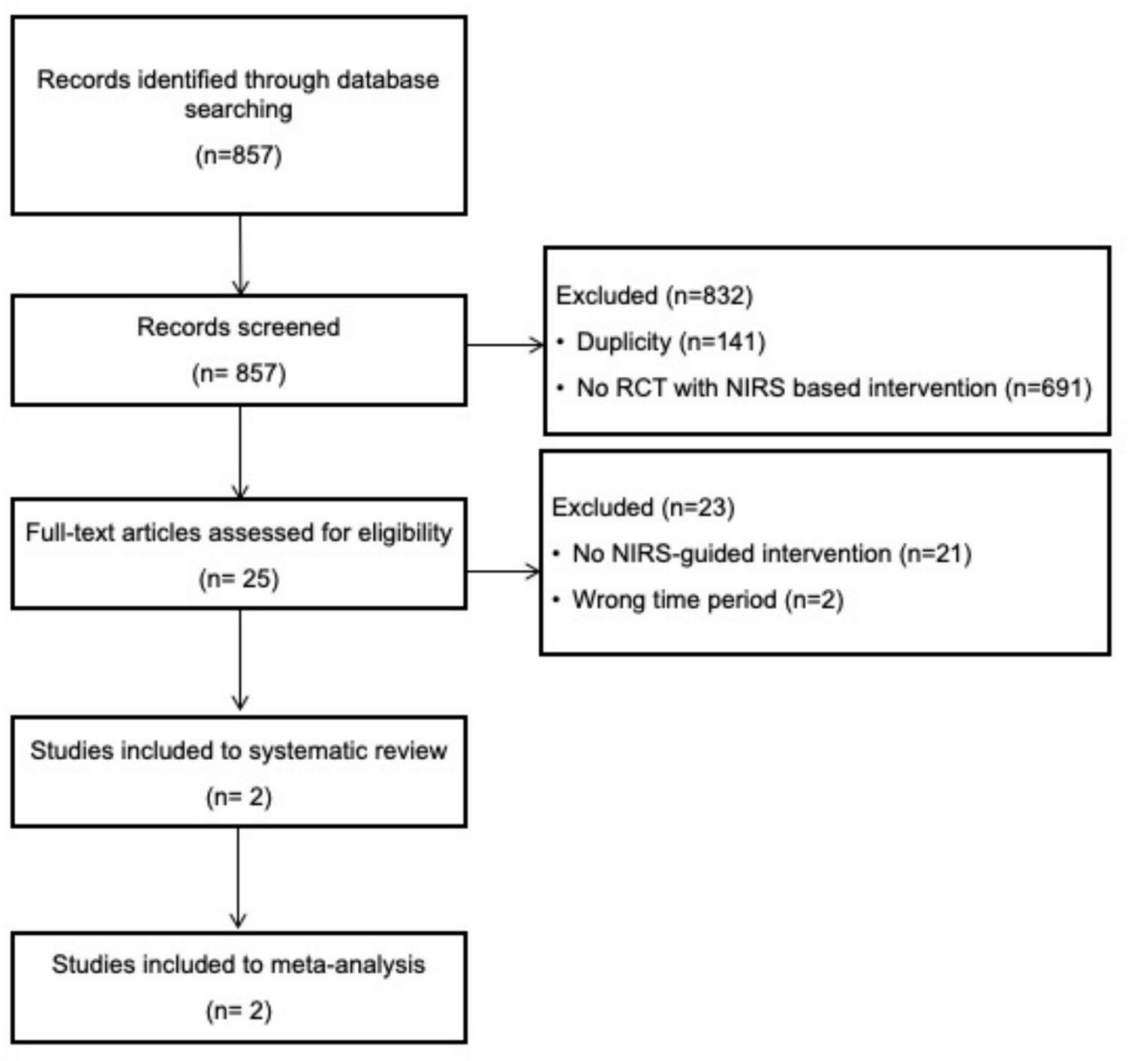
PRISMA flow diagram

The search yielded 857 records, of which 141 were removed as duplicates and 691 as no RCT with NIRS based intervention. After screening titles and abstracts 25 full-text articles were reviewed. Twenty-three studies were subsequently removed due to the wrong study design or timing of the interventions. Consequently, two RCTs were included in this review(24, 25). The PRISMA flow diagram is presented in Fig. 1.

### Characteristics of included studies

This systematic review included two RCTs (24, 25). The included studies compared interventions guided by NIRS-derived brain oxygenation measurements to the standard guideline-based care during neonatal resuscitation in the delivery room. In total, 667 preterm infants born at less than 34 weeks gestation were enrolled, with the majority being less than 32 weeks’ gestation. Characteristics of the included studies are shown in Table 1, and baseline characteristics of included preterm infants are displayed in Table 2.

**Table 1:** Characteristics of the included studies; RCT= randomized controlled trial

Study	Study design	NIRS-guided	Standard care	Inclusion criteria	Exclusion criteria
Pichler 2016 (24)	Two-center pilot feasibility RCT	n = 30	N = 30	<34 week’s gestation	Decision not to providefull life support,congenital malformation
Pichler 2023 (25)	Multicenter, multinational, phase III RCT	n = 304	n = 303	<32 week’s gestation	Decision not to providefull life support,congenital malformation

**Table 2:** Demographic data of included preterm infants, data are presented as mean ± SD, median (IQR), or n (%)

	**Pichler 2016**(24)		**Pichler 2023** (25)		**Pooled data**		
	NIRS- guided *n* = 30	Standard care *n* = 30	NIRS- guided *n* = 304	Standard care *n* = 303		NIRS- guided *n* = 334	Standard care *n* = 333
Gestational age (week)	29.8 ± 3.0	29.2 ± 2.9	28.9 (26.9–30.6)	28.6 (26.6–30.6)		29.0 (26.9–30.7)	28.6 (26.6–30.6)
Birth weight (grams)	1351 ± 540	1321 ± 467	1123 (860–1405)	1075 (820–1360)		1150 (850–1446)	1080 (829–1413)
Apgar 1	6 (4–8)	7 (5–8)	7 (5–8)	7 (5–8)		7 (5–8)	7 (5–8)
Apgar 5	8 (7–9)	8 (6–9)	8 (7–9)	8 (7–9)		8 (7–9)	8 (7–9)
Apgar 10	9 (8–9)	9 (8–9)	9 (8–9)	9 (8–9)		9 (8–9)	9 (8–9)
Male	16 (53.3%)	16 (53.3%)	148 (49.0%)	171 (56.8%)		164 (49.1%)	187 (56.2%)

### Study protocol and interventions

The interventions in the included studies, which were guided by CrSO2 measurements (24, 25), aimed to optimize cerebral oxygen delivery and perfusion. In the NIRS-guided group CrSO_2_ monitoring was visible to the clinical team, and additional supplemental oxygen or respiratory support were provided when preterm infants’ CrSO2 levels were outside the target range (12). If SpO2 remained within the target range but CrSO2was below the 10 th centile, FiO2 was increased, or respiratory support was started/increased (24, 25). If CrSO2 was above the 10 th centile for over 60 s or above the 90 th centile, FiO2 was reduced, or respiratory support was adjusted. The interventions were applied in addition to the standard resuscitation care (24, 25). In the larger RCT (25), intravenous fluids (10 mL/kg) were considered, if there was a history of blood loss or clinical signs of blood loss. The standard care group received standard resuscitation care according to current guidelines, and CrSO2 values were not visible to the clinical team (6, 7).

### Quality of individual studies

**Fig. 2:**
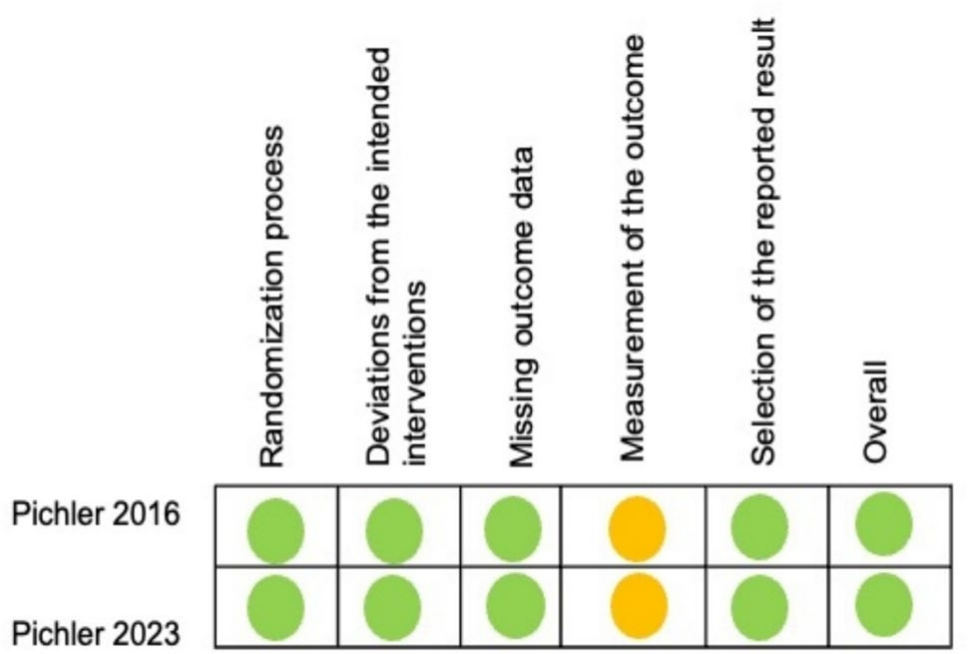
Assessment of risk bias for primary outcome

The included studies were judged to have a low risk of bias across most domains, including randomization, allocation concealment, and selective reporting. The assessment of potential sources of bias is presented in Fig. 2. The risk of bias of the included studies was evaluated using the Cochrane Collaboration Tool (21). Both studies reported an adequate method of allocation concealment. In the closed group, the resuscitation team was blinded to the CrSO2 values, with a member of the research team documenting the values. In the open group, no blinding was possible due to the nature of the intervention. Although the resuscitation team and healthcare providers were aware of the group assignments, the risk of detection bias can be described as low, since the outcomes were assessed using objective criteria. No other sources of risk were identified in the included studies.

### Bayesian-analysis

#### Primary outcome

**Fig. 3:**
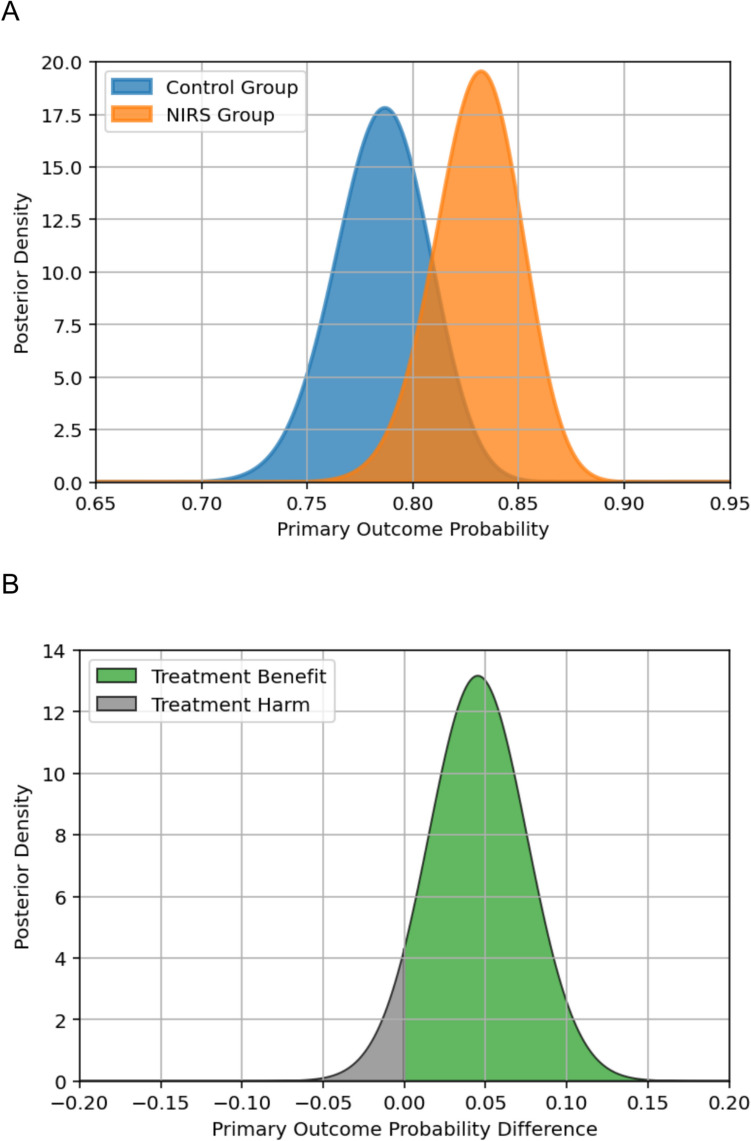
**A** Posterior density for primary outcome probability. **B** Posterior density for primary outcome probability difference (NIRS-guided group)

A Bayesian statistical analysis was performed to evaluate the degree of belief in the primary outcome of survival without cerebral injury. The posterior mean estimate for control group was 0.785, 95% credible interval (CI) [0.740, 0.827]. For the NIRS-guided group, these values corresponded to 0.830, 95% CI [0.789, 0.869], respectively. The mean difference was 0.045, 95% CI [− 0.014, 0.105]. The probability that the NIRS-guided interventions were more effective than routine care was 93%, suggesting high confidence in the direction of survival without cerebral injury (Fig. 3).

#### Secondary outcomes

The included studies additionally reported on secondary outcome measures including mortality and cerebral injury independently, and the incidence of necrotizing enterocolitis, retinopathy of prematurity, and bronchopulmonary dysplasia (Table 3).

**Table 3:** Bayesian analysis for primary and secondary outcome parameter

	Standard care (***n*** = 333)			NIRS-guided group (***n*** = 334)			Difference		Probability of treatment superiority
	*n*	Mean	95% CI	*n*	Mean	95% CI	Mean	95% CI	
Survival without cerebral injury	262	0.785	[0.740, 0.827]	278	0.830	[0.789, 0.869]	0.045	[− 0.014, 0.105]	0.932
Primary outcome measures									
Mortality	18	0.057	[0.035, 0.084]	12	0.039	[0.021, 0.062]	− 0.018	[− 0.051, 0.014]	0.867
IVH, any grade	58	0.176	[0.137, 0.219]	44	0.134	[0.100, 0.172]	− 0.042	[− 0.097, 0.012]	0.935
no IVH	275	0.824	[0.781, 0.863]	290	0.866	[0.828, 0.900]	0.042	[− 0.012, 0.097]	0.935
IVH I + II	41	0.125	[0.092, 0.163]	31	0.095	[0.066, 0.129]	− 0.030	[− 0.078, 0.017]	0.895
IVH III + IV	17	0.054	[0.032, 0.080]	13	0.042	[0.023, 0.065]	− 0.012	[− 0.044, 0.020]	0.771
Cystic PVL	3	0.012	[0.003, 0.026]	8	0.027	[0.012, 0.047]	0.015	[− 0.005, 0.037]	0.072
Secondary morbidities									
NEC	18	0.057	[0.035, 0.084]	18	0.057	[0.034, 0.084]	0.000	[− 0.035, 0.035]	0.504
ROP	34	0.104	[0.074, 0.139]	38	0.116	[0.084, 0.152]	0.012	[− 0.036, 0.059]	0.315
BPD	60	0.182	[0.143, 0.225]	59	0.179	[0.140, 0.221]	− 0.004	[− 0.062, 0.055]	0.547

IVH = intraventricular hemorrhage, PVL= periventricular leukomalacia, NEC= necrotizing enterocolitis, ROP= retinopathy of the prematurity, BPD= bronchopulmonary dysplasia

## Discussion

In this systematic review, we aimed to synthesize the existing evidence on the use of CrSO2 monitoring to guide neonatal resuscitation in preterm infants after birth. The individual patient data meta-analysis using the Bayesian method indicated a 93% probability for treatment superiority in the NIRS-guided group, with a mean improvement in survival without cerebral injury of 4.5%. The results showed that using NIRS to guide interventions during preterm infant resuscitation, aiming for target CrSO_2_ values, may improve survival without major cerebral injury, compared to standard care.

The COSGOD I/II pilot trial (24) showed that the use of NIRS in addition to routine monitoring to guide medical support during neonatal transition resulted in a 55% relative reduction in the risk of cerebral hypoxia. Subsequently, the COSGOD III study was conducted (25). Although the COSGOD III study reported an increased rate of survival without cerebral injury and a decreased risk of mortality in the intervention group, these findings did not reach statistical significance using the traditional frequentist method (25).

Similarly, the SafeboosC-II trial examined the use of cerebral oxygenation monitoring to guide treatment in preterm infants during their first 72 h after birth (26). This trial demonstrated a reduction in the burden of cerebral hypoxia in the intervention group compared to the control group. Additionally, it observed trends towards a decrease in severe brain injury and all-cause mortality. Consequently, the researchers conducted the larger SafeboosC-III trial, a multicenter, multinational RCT, with the primary outcome of death or severe brain injury (27). However, this larger trial found that the CrSO2 monitoring approach showed no difference in the occurrence of these critical outcomes compared to standard care.

The results of the COSGOD and SafeboosC studies collectively suggest that while the use of CrSO2 monitoring may provide valuable real-time information about brain oxygenation, its ability to meaningfully improve survival and reduce cerebral injury in preterm infants seems to be poor.

This prompts the question of whether there is no meaningful difference in outcome, or if the pathophysiological complexity underlying conditions like IVH and subsequent cerebral injury have been insufficiently addressed by the treatment protocols and statistical approaches used thus far. Addressing these underlying mechanisms, which involve numerous variables and pathways, may be the key to guide future research and developing more effective interventions to improve outcomes for preterm infants. Additionally, the present analysis of individual outcome measures within the primary outcome of this meta-analysis revealed a high probability (93.5%) of treatment superiority in the NIRS-guided group for IVH of any grade, with a mean improvement of 4.2%. Conversely, there was a higher probability for cystic PVL in the NIRS group, although the difference between the two groups was only 1.5%, suggesting questionable clinical relevance. However, an examination of the absolute numbers for cystic PVL, retinopathy of prematurity, necrotizing enterocolitis, and bronchopulmonary dysplasia indicates that NIRS-guided preterm resuscitation may not significantly influence these neonatal morbidities, and the low sample sizes for these diagnoses must be considered.

Within the last years frequentist statistics, which uses p-values to indicate statistical significance have come under scrutiny, especially when trying to translate results to clinical practice (28). The call for using Bayesian analysis, a different statistical approach, is growing in popularity(15, 17, 29). Bayesian analysis is a fundamentally different approach to statistical inference compared to the traditional frequentist paradigm. While frequentist analysis examines the likelihood of the observed data if the null hypothesis is true, in contrast, Bayesian analysis directly calculates the probability of the hypothesis being true given the observed data (16, 17). This may provide a more intuitive understanding of the results for clinical decision-making. Several methodological publications(15–17), such as the checklist provided by Ferreira et al. (18), have offered valuable guidance on the six steps to interpret clinical trial results analyzed using Bayesian methods (18, 30). Bayesian analysis allows researchers to formally incorporate prior knowledge or beliefs about the treatment effect into the analysis, the results of Bayesian analyses can be more intuitively understood by clinicians and decision-makers compared to traditional statistical measures (17, 29). A multidisciplinary team approach, involving clinicians, statisticians, and/or mathematicians, is crucial for reporting the results. It was suggested that researchers should be trained in the use of Bayesian methods and that the use of Bayesian statistics should be encouraged by editorial boards (30).

In the present meta-analysis, the Bayesian analysis demonstrated a high degree of confidence (92% probability) that the CrSO2-guided interventions improved the primary outcome of survival without cerebral injury. Considering the results of the Bayesian statistics in this review a clinically meaningful advantage for the individual preterm infant can be assumed. The positive effect of CrSO2-guided intervention could be explained by the ability of this approach to enable more nuanced adjustments in oxygen delivery and hemodynamic support, compared to reliance on standard clinical assessment methods alone. This appears to be most prominent during the crucial first minutes after birth. 

### Strengths and Limitations

This systematic review has some limitations. The small number of included studies constrains the power and precision of the meta-analysis. Additionally, both included studies originated from the same center, which may be a strength due to similar study design, but could also indicate a higher risk of similar biases across the two studies. However, both included studies were performed on multiple sites. Another limitation might be that both trials utilized cranial ultrasound, rather than the more precise MRI. However, cranial ultrasound is the routine bedside method in most neonatal intensive care units to evaluate cerebral injuries. Furthermore, while it is possible that minor injuries may have been overlooked by ultrasound, the authors believe that this should be similar in both groups and therefore should not have caused a significant systematic difference between the groups. The meta-analysis included two RCTs that enrolled preterm infants, with the larger trial focusing exclusively on infants born before 32 weeks of gestation. Both RCTs used CrSO_2_ reference ranges derived from a study that did not involve infants born before 32 weeks (12). Meanwhile, new reference ranges specifically for infants under 32 weeks have since been developed, and these exhibit lower values compared to the ranges utilized in the two RCTs (13). However, in both trials in both groups, the same centiles were used and therefore should also not have caused a significant systematic difference between the groups. A strength of the present review is that it utilized a robust Bayesian statistical approach, which provides a more nuanced understanding of the potential benefits of NIRS-guided preterm infant resuscitation compared to the classical frequentist meta-analysis.

## Conclusion

This systematic review suggests that CrSO2-guided interventions may offer a meaningful advantage in preterm infant resuscitation after birth, with a 4.5% higher rate of survival without severe brain injury compared to standard care. The Bayesian analysis indicates a high probability of a clinically important benefit for survival without cerebral injury and IVH of any grade alone.

Given the substantial challenges of preterm birth, any intervention enhancing the likelihood of survival without brain injury would be hugely impactful, for the infant, family, and healthcare system. Consideration should be given to the use of CrSO2 in preterm infants to guide neonatal stabilization at birth.

## Supplementary Information

Below is the link to the electronic supplementary material.

ESM 1 (PDF 168 KB)

## Data Availability

No datasets were generated or analysed during the current study.

## Appendix

### Search terms:

(neonate or newborn) AND.

(cerebral oximetry or cerebral regional oxygenation or near infrared spectroscopy or NIRS) AND.

(delivery room or resuscitation or transition or after birth) AND.

(intervention or care or treatment).

## Abbreviations

CrSO_2_: Cerebral tissue oxygen saturation
ECG: Electrocardiogram
IVH: Intraventricular hemorrhage
NIRS: Near-infrared spectroscopy
PVL: Periventricular leukomalacia
RCT: Randomized controlled trial
SpO_2_: Peripheral arterial oxygen saturation

## Declarations

### AI statement

During the preparation of this work, the authors did not use artificial intelligence.
