# Supplementary material for: Brain oxygenation monitoring during neonatal stabilization and resuscitation and its potential for improving preterm infant outcomes: a systematic review and meta-analysis with Bayesian analysis
Source: Eur J Pediatr. 2025 Apr 21;184(5):305. doi: 10.1007/s00431-025-06138-0 (PMC12011959; doi:10.1007/s00431-025-06138-0)
Supplement: Supplementary file 1 — (PDF 168 KB) [file 431_2025_6138_MOESM1_ESM.pdf]

COSGOD: Bayesian Analysis

Thomas Suppan

January 11, 2025

Contents

1 Bayesian Formulation **2**
1.1 Observations 2
1.2 Parameters 2
1.3 Hierarchical Model for Meta Analyses 2
1.4 Likelihood Function 3
1.5 Prior Distribution 3
1.6 Posterior Distribution 4

2 Bayesian Inference **4**
2.1 Moments 4
2.2 Quantiles 4
2.3 Hypothesis Testing 4
2.3.1 Numerical Integration 5

1 Bayesian Formulation

1.1 Observations

The observation for patient i and study j is

$$y_{i,j} = \begin{cases} 1 & \text{if primary/secondary outcome} \\ 0 & \text{if no primary/secondary outcome} \end{cases}. \tag{1.1}$$

The observations are therefore binary, i.e. $y_{k,l} \in \{0, 1\}$. In the following, $\mathbf{y}_j$ denotes a vector holding the N_j observations of study j.

1.2 Parameters

The parameters of interest are the probabilities to observe the primary/secondary outcome of the individual studies θ_j as well as the overall probability to observe the primary/secondary outcome θ, i.e. the pooled effect size of the meta analysis.

1.3 Hierarchical Model for Meta Analyses

Given a number of L studies, the posterior distribution for the parameters θ_j for $j = 1, \dots L$ and θ given the observations $\mathbf{y}_j$ for $j = 1, \dots L$ is

$$p\left(\theta_1, \theta_2, \dots, \theta_L, \theta \middle| \mathbf{y}_1, \mathbf{y}_2, \dots, \mathbf{y}_L\right) = \frac{p\left(\mathbf{y}_1, \mathbf{y}_2, \dots, \mathbf{y}_L \middle| \theta_1, \theta_2, \dots, \theta_L, \theta\right) p\left(\theta_1, \theta_2, \dots, \theta_L, \theta\right)}{p\left(\mathbf{y}_1, \mathbf{y}_2, \dots, \mathbf{y}_L\right)} \tag{1.2}$$

The likelihood function is

$$p\left(\mathbf{y}_1, \mathbf{y}_2, \dots, \mathbf{y}_L \middle| \theta_1, \theta_2, \dots, \theta_L, \theta\right) = p\left(\mathbf{y}_1, \mathbf{y}_2, \dots, \mathbf{y}_L \middle| \theta_1, \theta_2, \dots, \theta_L\right) = \prod_{j=1}^{L} p\left(\mathbf{y}_l \middle| \theta_l\right) \tag{1.3}$$

since the observations do not directly depend on the pooled effect size θ. The observations depend on θ only via the prior distribution by

$$p\left(\theta_1, \theta_2, \dots, \theta_L, \theta\right) = p\left(\theta_1, \theta_2, \dots, \theta_L \middle| \theta\right) p\left(\theta\right), \tag{1.4}$$

which also models the statistical dependency between the studies.
In Bayesian meta analysis a Gaussian prior distribution of the form

$$p\left(\theta_1, \theta_2, \dots, \theta_L \middle| \theta\right) = \prod_{l=1}^{L} \mathcal{N}\left(\theta_l \middle| \theta, \sigma^2\right), \tag{1.5}$$

is used as prior distribution. Hereby, the variance σ^2 is the between study heterogeneity.
A special case in meta analyses is the fixed effect model, which is preferred for small numbers of included studies and/or if the studies stem from the same center. Hereby, the effect sizes θ_j are assumed to follow the same overarching distribution. For this purpose, the variance σ^2 is set to zero, i.e. $\sigma^2 = 0$. In the limit $\sigma^2 = 0$, the normal distribution becomes

$$\lim_{\sigma^2 \to 0} \mathcal{N}\left(\theta_l \middle| \theta, \sigma^2\right) = \delta\left(\theta_l - \theta\right), \tag{1.6}$$

where $\delta(\cdot)$ is the Dirac delta function. With this, all of the probability mass is concentrated at the point θ. The prior distribution becomes

$$p\left(\theta_1, \theta_2, \dots, \theta_L \middle| \theta\right) = \prod_{l=1}^{L} \delta\left(\theta_l - \theta\right) \tag{1.7}$$

and the resulting posterior distribution can be stated as

$$p\left(\theta_1, \theta_2, \ldots, \theta_L, \theta \middle| \mathbf{y}_1, \mathbf{y}_2, \ldots, \mathbf{y}_L\right) = \\ = \begin{cases} \frac{p\left(\mathbf{y}_1, \mathbf{y}_2, \ldots, \mathbf{y}_L \middle| \theta_1, \theta_2, \ldots, \theta_L, \theta\right) p(\theta)}{p(\mathbf{y}_1, \mathbf{y}_2, \ldots, \mathbf{y}_L)} & \text{if} \quad \theta_1 = \theta_2 = \cdots = \theta_L = \theta\,. \\ 0 & \text{else} \end{cases} \tag{1.8}$$

Thus, for the fixed effect model the posterior distribution is none zero only if all effect sizes are equal as it is assumed that there is one fixed pooled effect size. With this the posterior distribution can be reformulated as

$$\begin{aligned} p\left(\theta \middle| \mathbf{y}_1, \mathbf{y}_2, \ldots, \mathbf{y}_L\right) &= p\left(\theta \middle| \mathbf{y}_1, \mathbf{y}_2, \ldots, \mathbf{y}_L\right) \\ &= \frac{p\left(\mathbf{y}_1, \mathbf{y}_2, \ldots, \mathbf{y}_L \middle| \theta\right) p\left(\theta\right)}{p\left(\mathbf{y}_1, \mathbf{y}_2, \ldots, \mathbf{y}_L\right)}, \end{aligned} \tag{1.9}$$

with the single parameter θ.

1.4 Likelihood Function

Since the observation $y_{i,j}$ of the patient i and study j is a binary variable and a number of N_j patients is included in each study, the data is modelled by a binomial distribution. The conditional probability mass function of the observations $\mathbf{y}_j$, given the parameter θ is

$$p\left(\mathbf{y}_j \middle| \theta\right) = \binom{N_j}{M_j} \theta^{M_j} \left(1-\theta\right)^{N_j - M_j}, \tag{1.10}$$

where M_j is the number of patients for which the primary/secondary outcome is observed

$$M_j = \sum_{i=1}^{N_j} y_{i,j} \tag{1.11}$$

and $\binom{N_j}{M_j}$ is the binomial coefficient

$$\binom{N_j}{M_j} = \frac{N_j!}{M_j!\,(N_j - M_j)!}. \tag{1.12}$$

Thus, the likelihood function is given by

$$p\left(\mathbf{y}_1, \mathbf{y}_2, \ldots, \mathbf{y}_L \middle| \theta\right) = \prod_{j=1}^{L} \binom{N_j}{M_j} \theta^{M_j} \left(1-\theta\right)^{N_j - M_j} \tag{1.13}$$

1.5 Prior Distribution

As θ is a probability it can only take values in the range $0 \leq \theta \leq 1$. For this purpose a weakly-informative prior distribution can be formulated by a uniform probability density function of the form

$$p\left(\theta_l\right) = \begin{cases} 1 & \text{if} \quad 0 \leq \theta_l \leq 1 \\ 0 & \text{otherwise.} \end{cases}. \tag{1.14}$$

With this kind of prior distribution no particular value of θ is favoured within the range $0 \leq \theta_l \leq 1$ while restricting θ to feasible values.

1.6 Posterior Distribution

In the following, the observations of the L studies are summarized by $\mathbf{Y} = [\mathbf{y}_1, \mathbf{y}_2, \ldots, \mathbf{y}_L]$. Given the likelihood function $p(\mathbf{y}_1, \mathbf{y}_2, \ldots, \mathbf{y}_L|\theta) = p(\mathbf{Y}|\theta)$ and the prior distribution $p(\theta)$, the posterior distribution $p(\theta|\mathbf{y}_1, \mathbf{y}_2, \ldots, \mathbf{y}_L) = p(\theta|\mathbf{Y})$ can be formulated using Bayes' theorem

$$p\left(\theta|\,\mathbf{Y}\right) = \frac{p\left(\mathbf{Y}|\,\theta\right)p\left(\theta\right)}{p\left(\mathbf{Y}\right)}. \tag{1.15}$$

Hereby, the normalization constant $p(\mathbf{Y})$ is referred to as evidence, which is computed by the marginalization integral

$$p\left(\mathbf{Y}\right) = \int_{-\infty}^{\infty} p\left(\mathbf{Y}|\,\theta\right)p\left(\theta\right)\,\mathrm{d}\theta. \tag{1.16}$$

Thus, the posterior distribution is fully described.

2 Bayesian Inference

2.1 Moments

Having formulated the posterior distribution $p(\theta|\mathbf{Y})$, conclusions about the parameters of interest θ can be drawn. For this purpose, moments of $p(\theta|\mathbf{Y})$ are computed, such as the first moment, i.e. the conditional mean

$$\mu_\theta = \int_{-\infty}^{\infty} \theta p\left(\theta\,|\mathbf{Y}\right)\,\mathrm{d}\theta. \tag{2.1}$$

An uncertainty measure can be obtained by the evaluation of the second centralized moment of $p(\theta|\mathbf{Y})$, i.e. the variance

$$\sigma_\theta^2 = \int_{-\infty}^{\infty} \left(\theta - \mu_\theta\right)^2 p\left(\theta\,|\mathbf{Y}\right)\,\mathrm{d}\theta. \tag{2.2}$$

2.2 Quantiles

In addition, quantiles of the posterior distribution can be computed. For example, the p-quantile q_p of the parameter θ can be found by the solution of the integral equation

$$\int_{-\infty}^{q_p} p\left(\theta\,|\mathbf{Y}\right)\,\mathrm{d}\theta = p. \tag{2.3}$$

2.3 Hypothesis Testing

The aim is to test the hypothesis that the probability θ for the primary/secondary outcome is improved for a specific set of observations $\mathbf{Y}_1$ (NIRS group) compared to a second set of observations $\mathbf{Y}_0$ (control group). For this purpose, the posterior distributions $p_0(\theta_0|\mathbf{Y}_0)$ and $p_1(\theta_1|\mathbf{Y}_1)$ are formulated for both groups. Having formulated the posterior distributions $p_0(\theta_0|\mathbf{y}_1)$ and $p_1(\theta_1|\mathbf{Y}_1)$, the probability that the hypothesis holds can be evaluated, i.e. the probability that $\theta_1 > \theta_0$. For independent θ_0 and θ_1 the joint posterior distribution is given by

$$p\left(\theta_0, \theta_1|\,\mathbf{Y}_0, \mathbf{Y}_1\right) = p_0\left(\theta_0|\,\mathbf{Y}_0\right)p_1\left(\theta_1|\,\mathbf{Y}_1\right). \tag{2.4}$$

The probability that $\theta_1 > \theta_0$ can be evaluated by the integral

$$P(\theta_1 > \theta_0) = \int_{-\infty}^{\infty} \int_{-\infty}^{\theta_1} p_1(\theta_1, |\, \mathbf{Y}_1)\, p_0(\theta_0 |\, \mathbf{Y}_0)\ \mathrm{d}\theta_0 \mathrm{d}\theta_1. \tag{2.5}$$

Alternatively, the posterior density for the effect difference $\Delta\theta = \theta_1 - \theta_0$ can be formulated by

$$p(\Delta\theta |\, \mathbf{Y}_0, \mathbf{Y}_1) = \int_{-\infty}^{\infty} p_1(\theta_1 |\, \mathbf{Y}_1)\, p_0(\theta_1 - \Delta\theta |\, \mathbf{Y}_0)\ \mathrm{d}\theta_1 \tag{2.6}$$

If the hypothesis holds the difference $\Delta\theta$ is positive. $p(\Delta\theta|\mathbf{Y}_0, \mathbf{Y}_1)$ can be used to analyse statistical properties of $\Delta\theta$ by means of moments and quantiles. The probability $P(\theta_1 > \theta_0)$ corresponds to the area under $p(\Delta\theta|\mathbf{Y}_0, \mathbf{Y}_1)$ for positive values of $\Delta\theta$, i.e.

$$P(\theta_1 > \theta_0) = \int_{0}^{\infty} p(\Delta\theta |\, \mathbf{Y}_0, \mathbf{Y}_1)\ \mathrm{d}\Delta\theta. \tag{2.7}$$

2.3.1 Numerical Integration

Since most integral equation involved in Bayesian inference cannot be solved analytically, numerical techniques are applied. For low dimensional problems numerical integration can be used. Hereby, integrals over a function $f(x)$ are approximated by a finite sum over the function values $f(x_n)$ evaluated at discrete points x_n, i.e.

$$\int_{a}^{b} f(x)\ \mathrm{d}x \approx \sum_{n=1}^{N} f(x_n)\, \Delta x, \tag{2.8}$$

with

$$\Delta x = \frac{b-a}{N}. \tag{2.9}$$

For decreasing Δx, i.e. increasing N this sum converges to the integral, i.e.

$$\lim_{\Delta x \to 0} \sum_{n=1}^{N} f(x_n)\, \Delta x = \int_{a}^{b} f(x)\ \mathrm{d}x. \tag{2.10}$$
